# Supplementary material for: Relic populations of Fukomys mole-rats in Tanzania: description of two new species F. livingstoni sp. nov. and F. hanangensis sp. nov
Source: PeerJ. 2017 Apr 27;5:e3214. doi: 10.7717/peerj.3214 (PMC5410139; doi:10.7717/peerj.3214)

Figure S1. Craniometric points used to take linear measurements of the skull, with numbers referring to the position of the caliper jaws when taking the measurement as follows: M1 greatest length of skull (between anteriormost point of the incisor to the posteriormost point of the supraoccipital processes); M2 condylobasal length (least distance from the posteriormost projections of the exoccipital condyles to a line connecting the anteriormost projections of the premaxillary bones); M3 henselion-basion length (distance between the posterior margin of the palate and the posteriormost margin of the alveolus of the incisors); M4 henselion-palation length (distance between the anteriormost part of the foramen magnum and the posteriormost margin of the alveolus of the incisors); *M5 length of palatal incisive foramen (= palatal foramen) not recorded*; M6 length of diastema (distance between the anterior border of the alveolus of M1 and the posterior border of the alveolus of the upper incisor); M7 distance between the anterior border of the alveolus of M1 and the foremost edge of the upper incisor; M8 smallest interorbital breadth; M9 zygomatic breadth on the zygomatic process of the squamosal; M10 smallest palatal breadth between first upper molars; M11 length of upper cheekteeth (distance between the anterior border of the alveolus of M1 and the posterior border of the alveolus of M4); M12 breadth of upper dental arch: greatest breadth across first upper molars; M13 greatest breadth of first upper molar; M14 smallest breadth of zygomatic plate: distance taken in a plane parallel to the occlusal surface of the upper molar-row; M15 greatest breadth of nasals; M16 greatest length of nasals; M17 length of lower cheekteeth: distance between the anterior border of the alveolus of M1 and the posterior border of the alveolus of M4; M18 greatest breadth of the choanae; M19 length of auditory bulla (the protruding part of the bony Eustachian tube); M20 greatest breadth of braincase; M21 depth of upper incisors (perpendicular on length axis of tooth); M22 mediosagittal projection of rostrum height at anterior border of first upper molars; M23 greatest rostrum breadth (in front of zygomatic plates); M24 distance between the extreme points of the coronoid and the angular processes of the mandibular. Based on Verheyen *et al.* (1996).

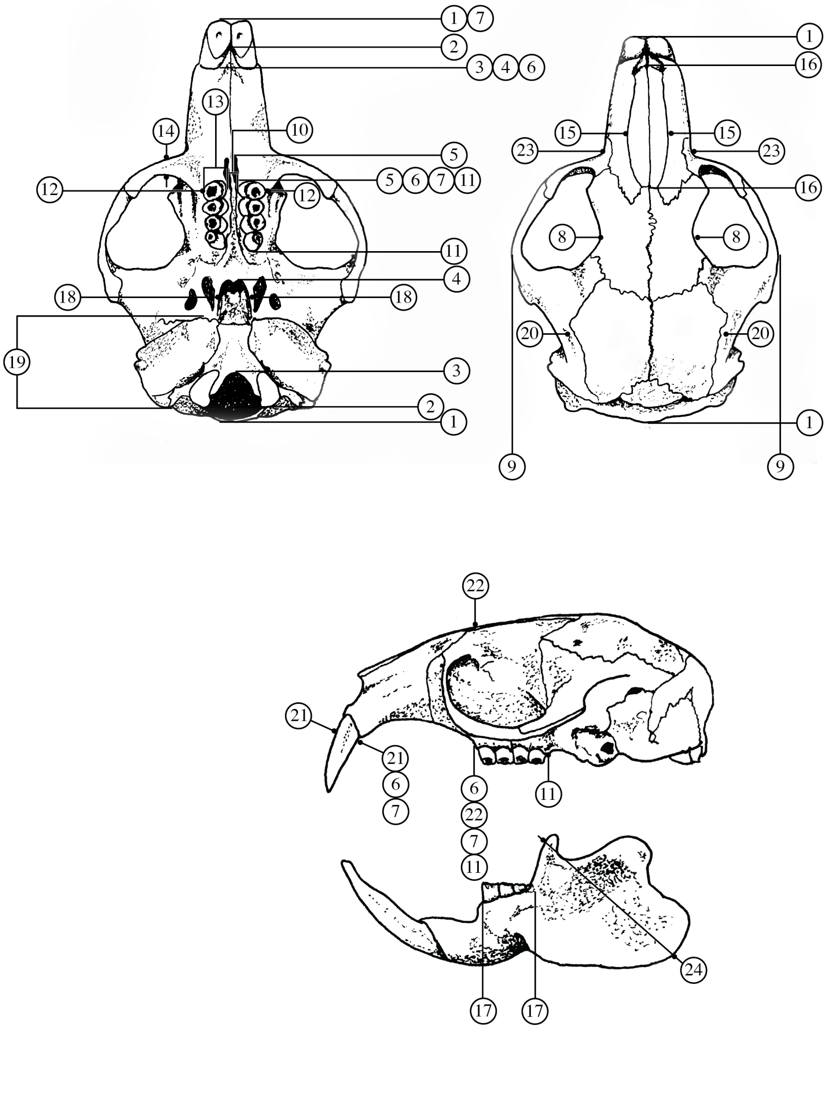

Supplement: Figure S1 — Craniometric points used to take linear measurements of the skull, with numbers referring to the position of the caliper jaws when taking the measurement as follows: M1 greatest length of skull (between anteriormost point of the incisor to the posteriormost point of the supraoccipital processes); M2 condylobasal length (least distance from the posteriormost projections of the exoccipital condyles to a line connecting the anteriormost projections of the premaxillary bones); M3 henselion-basion length (distance between the posterior margin of the palate and the posteriormost margin of the alveolus of the incisors); M4 henselion-palation length (distance between the anteriormost part of the foramen magnum and the posteriormost margin of the alveolus of the incisors); M5 length of palatal incisive foramen ( = palatal foramen) not recorded; M6 length of diastema (distance between the anterior border of the alveolus of M1 and the posterior border of the alveolus of the upper incisor); M7 distance between the anterior border of the alveolus of M1 and the foremost edge of the upper incisor; M8 smallest interorbital breadth; M9 zygomatic breadth on the zygomatic process of the squamosal; M10 smallest palatal breadth between first upper molars; M11 length of upper cheekteeth (distance between the anterior border of the alveolus of M1 and the posterior border of the alveolus of M4); M12 breadth of upper dental arch: greatest breadth across first upper molars; M13 greatest breadth of first upper molar; M14 smallest breadth of zygomatic plate: distance taken in a plane parallel to the occlusal surface of the upper molar-row; M15 greatest breadth of nasals; M16 greatest length of nasals; M17 length of lower cheekteeth: distance between the anterior border of the alveolus of M1 and the posterior border of the alveolus of M4; M18 greatest breadth of the choanae; M19 length of auditory bulla (the protruding part of the bony Eustachian tube); M20 greatest breadth of braincase; M21 de [file peerj-05-3214-s001.pdf]
