# Supplementary material for: Relic populations of Fukomys mole-rats in Tanzania: description of two new species F. livingstoni sp. nov. and F. hanangensis sp. nov
Source: PeerJ. 2017 Apr 27;5:e3214. doi: 10.7717/peerj.3214 (PMC5410139; doi:10.7717/peerj.3214)

Figure S2. Positions of landmarks used in the shape analysis of the dorsal (a) and ventral (b) skulls. Dorsal landmarks: 1, distal tip of the median line; 2, anterior junction of nasal and premaxilla; 3, junction of rostrum and zygomatic process; 4, junction of jugal and zygomatic process; 5, orbital junction of zygomatic process and frontal; 6, posterior junction of jugal with squamosal; 7, orbital junction of frontal and squamosal; 8, intersection of parietal, squamosal and frontal; 9, right anterolateral tip of the parietal bone; 10, point of narrowest inflection of squamosal viewed from above; 11, anterior tip of the interparietal bone; 12, cross point between the median line and the line which connects left and right anterolateral tip of the interparietal bone; 13, posterior tip of premaxilla; 14, posterior junction of nasal and premaxilla; 15, posterior junction of nasals. Ventral landmarks: 1, distal tip of the premaxilla at the midline; 2, ; lateral extent of the premaxilla at the incisor. 3, anteriormost section of the zygomatic process at the junction of the premaxilla; 4, junction of jugal and zygomatic process; 5, posterior junction of jugal with squamosal; 6, posterolateral edge of the squamosal; 7, junction of squamosal and auditory bulla; 8, lateral tip of auditory bulla; 9, posterior tip of auditory bulla at the junction with the occipital; 10, lateral extent of the foramen magnum; 11, anterior mid-point of the foramen magnum; 12, anterior edge of the occipital at the midline; 13, junction of occipital, basioccipital and auditory bulla; 14, anterior mid-point of the choanae ; 15, posterior border of the alveolus of M^4; 16, anterior border of the alveolus of M^1; 17, anterior tip of the palatal foramen.

(a)

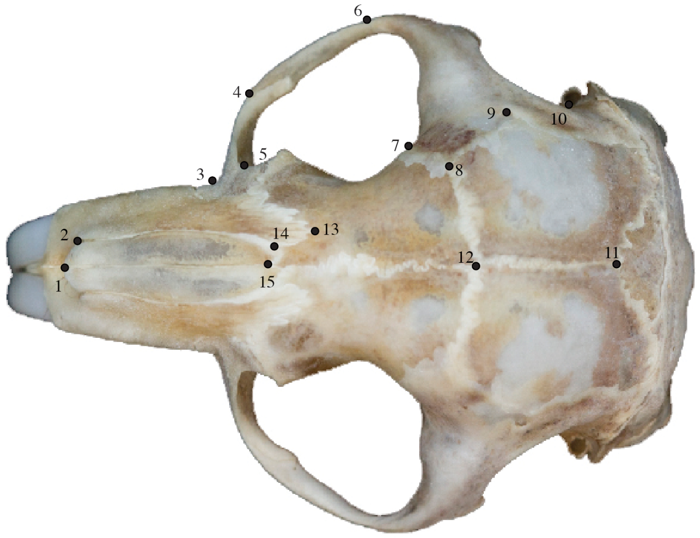

(b)

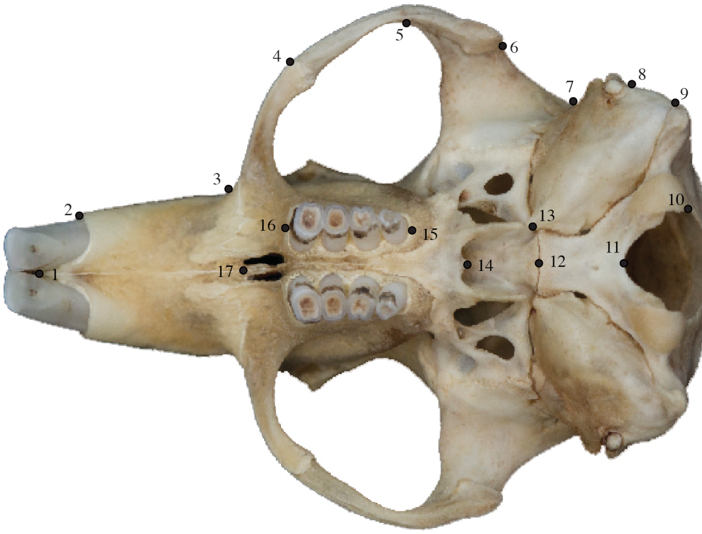

Supplement: Figure S2 — Positions of landmarks used in the shape analysis of the dorsal (a) and ventral (b) skulls. Dorsal landmarks: 1, distal tip of the median line; 2, anterior junction of nasal and premaxilla; 3, junction of rostrum and zygomatic process; 4, junction of jugal and zygomatic process; 5, orbital junction of zygomatic process and frontal; 6, posterior junction of jugal with squamosal; 7, orbital junction of frontal and squamosal; 8, intersection of parietal, squamosal and frontal; 9, right anterolateral tip of the parietal bone; 10, point of narrowest inflection of squamosal viewed from above; 11, anterior tip of the interparietal bone; 12, cross point between the median line and the line which connects left and right anterolateral tip of the interparietal bone; 13, posterior tip of premaxilla; 14, posterior junction of nasal and premaxilla; 15, posterior junction of nasals. Ventral landmarks: 1, distal tip of the premaxilla at the midline; 2, ; lateral extent of the premaxilla at the incisor. 3, anteriormost section of the zygomatic process at the junction of the premaxilla; 4, junction of jugal and zygomatic process; 5, posterior junction of jugal with squamosal; 6, posterolateral edge of the squamosal; 7, junction of squamosal and auditory bulla; 8, lateral tip of auditory bulla; 9, posterior tip of auditory bulla at the junction with the occipital; 10, lateral extent of the foramen magnum; 11, anterior mid-point of the foramen magnum; 12, anterior edge of the occipital at the midline; 13, junction of occipital, basioccipital and auditory bulla; 14, anterior mid-point of the choanae ; 15, posterior b [file peerj-05-3214-s002.pdf]
