# Supplementary material for: Relic populations of Fukomys mole-rats in Tanzania: description of two new species F. livingstoni sp. nov. and F. hanangensis sp. nov
Source: PeerJ. 2017 Apr 27;5:e3214. doi: 10.7717/peerj.3214 (PMC5410139; doi:10.7717/peerj.3214)

Figure S3. Artists impressions (drawn from specimens). (a) *Fukomys livingstoni* and (b) *Fukomys hanangensis*. Artwork by Rebecca Gelernter (www.nearbirdstudios.com), who retains the copyright on this image (used with permission). Not to scale.

(a)

(b)

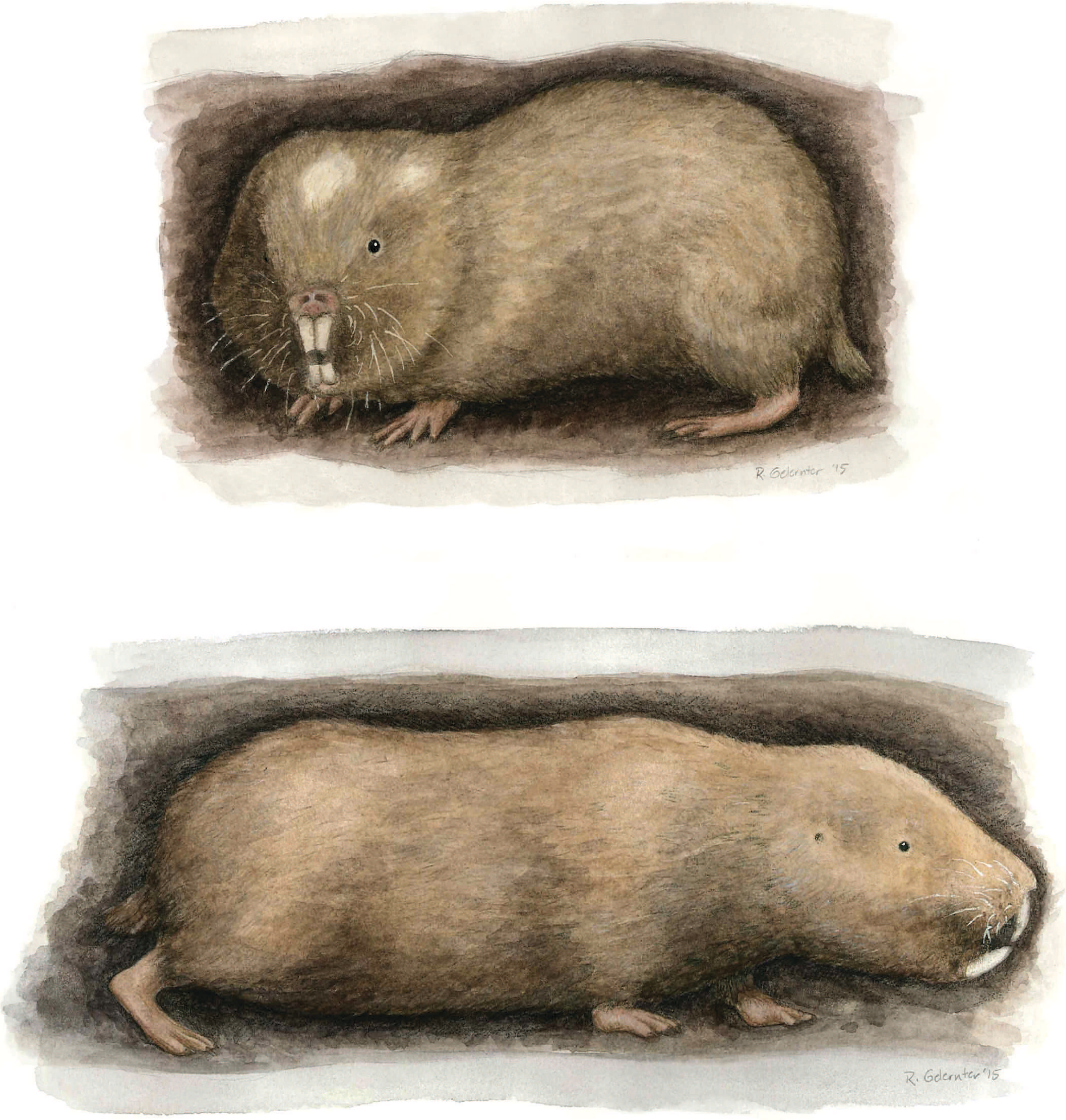

Supplement: Figure S3 — (a) Fukomys livingstoni and (b) Fukomys hanangensis. Artwork by Rebecca Gelernter (www.nearbirdstudios.com), who retains the copyright on this image (used with permission). Not to scale. [file peerj-05-3214-s003.pdf]
