# Supplementary material for: Relic populations of Fukomys mole-rats in Tanzania: description of two new species F. livingstoni sp. nov. and F. hanangensis sp. nov
Source: PeerJ. 2017 Apr 27;5:e3214. doi: 10.7717/peerj.3214 (PMC5410139; doi:10.7717/peerj.3214)

Figure S4. Scatterplots showing comparative measurements of (a) craniometric (greatest skull width at zygomatic arch against greatest skull length) and (b) morphometric data (body length against body weight) for a range of south-central African *Fukomys* species. Individual points are means with horizontal and vertical error bars indicating sample ranges. Species and sample sizes as indicated (squares denote males, circles females, diamond symbol, sexes unknown). All data from Kingdom *et al*. (2013) except *F. vandewoestinjneae* (Van Daele *et al*., 2013).

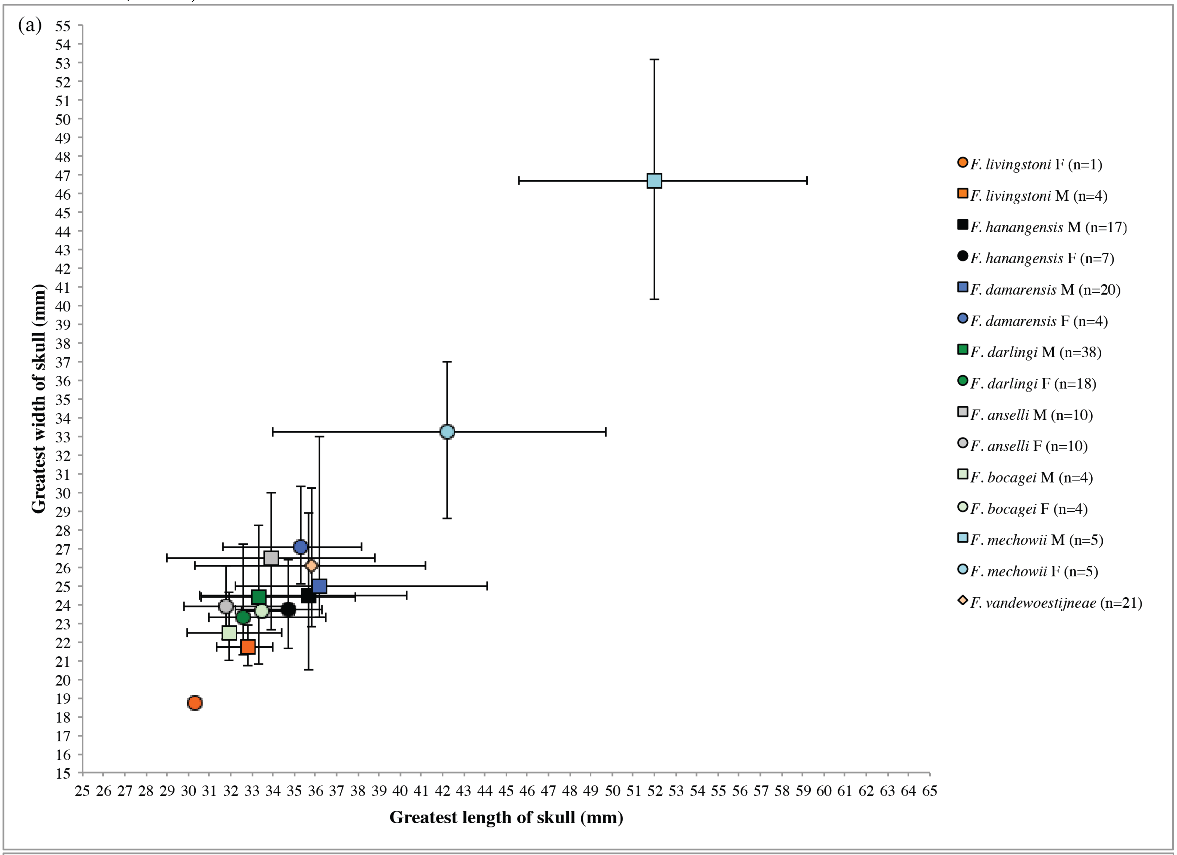

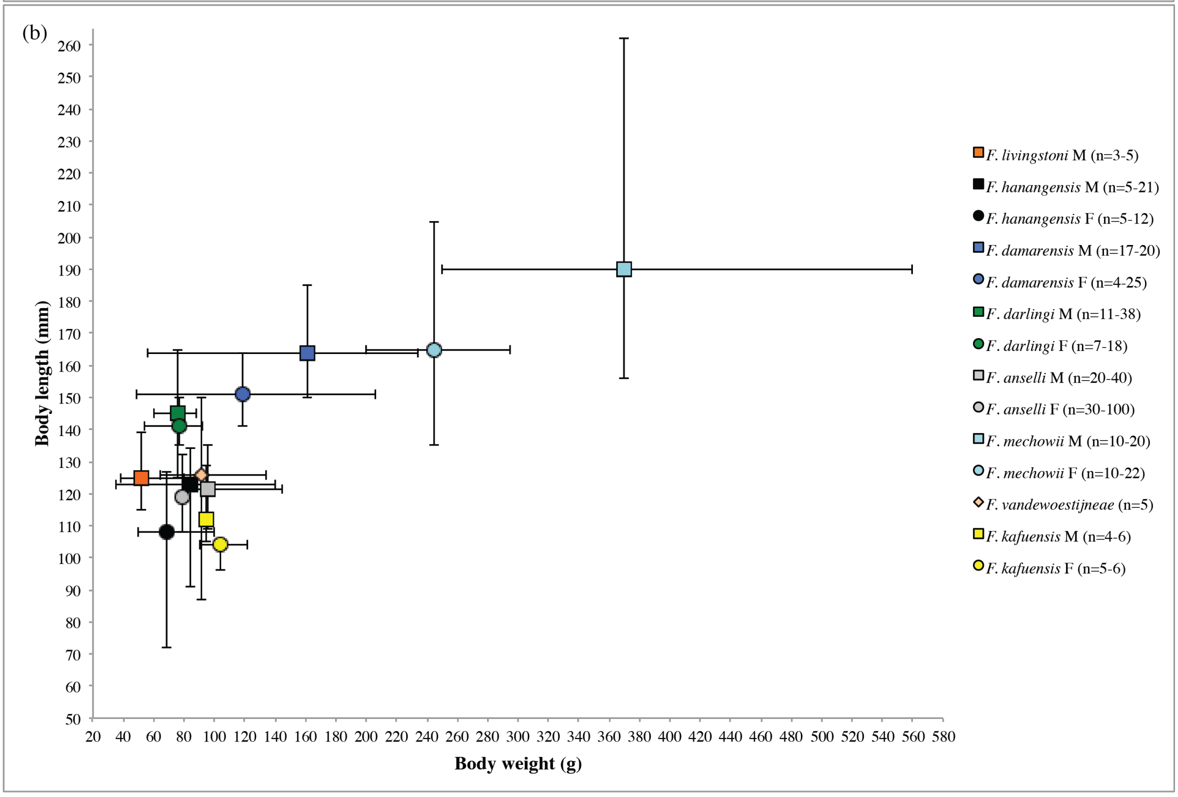

Supplement: Figure S4 — (a) Craniometric (greatest skull width at zygomatic arch against greatest skull length) and (b) morphometric data (body length against body weight) for a range of south-central African Fukomys species. Individual points are means with horizontal and vertical error bars indicating sample ranges. Species and sample sizes as indicated (squares denote males, circles females, diamond symbol, sexes unknown). All data from Kingdom et al. (2013) except F. vandewoestinjneae (Van Daele et al., 2013). [file peerj-05-3214-s004.pdf]
