# Supplementary material for: Relic populations of Fukomys mole-rats in Tanzania: description of two new species F. livingstoni sp. nov. and F. hanangensis sp. nov
Source: PeerJ. 2017 Apr 27;5:e3214. doi: 10.7717/peerj.3214 (PMC5410139; doi:10.7717/peerj.3214)
Supplement: Table S1 — Individual and mean ± SEM (mm) head and body length (H&B), tail, and hind-foot lengths, (Hft) together with craniometric measurements M1 to M24, as in Figure S1. [file peerj-05-3214-s005.pdf]

Table S1. Morphometric and craniometric data. Individual and mean ± SEM (mm) head and body length (H&B), tail, and hind-foot lengths, (Hft) together with craniometric measurements M1 to M24, as in Fig. S1.

Species	Sample no QMUL	Sample type	Location	Colony	Sex	bw (g)	Age class	Specimen	H&B	Tail	Hft (left)	Hft (right)	M1	M1/M9	M2	M3	M4	M6	M7	M8	M9	M10	M11	M12	M13	M14	M15	M16	M17	M18	M19	M20	M21	M22	M23	M24
F. hanangensis	3906	QM sample	Hanang	na	na	na	2	skull & tissue					34.97	1.55	31.58	26.72	18.50	10.66	13.90	8.57	22.53	1.33	5.67	5.17	1.84	1.17	2.91	13.07	6.56	2.56	7.44	14.42	1.88	9.72	6.67	14.57
F. hanangensis	3907	QM sample	Hanang	na	na	na		tissue only																												
F. hanangensis	3928	QM sample	Hanang	na	na	na	2	skull & tissue					34.37	1.51	30.88	26.96	17.81	9.58	13.33	8.40	22.79	0.83	6.42	5.25	1.81	1.45	3.16	11.87	6.28	2.10	8.08	13.41	1.83	10.00	6.58	15.46
F. hanangensis	4303	Paratype	Hanang	1	M	40		whole animal	90.6	10.0	21.5	21.3																								
F. hanangensis	4304	QM sample	Hanang	1	F	50		whole animal	72.2	9.5	21.9	22.0																								
F. hanangensis	4305	QM sample	Hanang	2	M	120		tissue only																												
F. hanangensis	4306	QM sample	Hanang	2	na	na		tissue only																												
F. hanangensis	4307	QM sample	Hanang	2	na	50		tissue only																												
F. hanangensis	4308	Holotype	Hanang	1	BrF	62		whole animal	111.0	12.2	23.1	23.1																								
F. hanangensis	4309	Paratype	Mbulu	2	F	57	2	skull & tissue					33.91	1.48	29.46	25.64	17.50	9.49	13.21	9.14	22.86	1.06	6.15	5.71	2.12	1.59	3.58	11.08	6.44	2.50	8.04	13.92	1.70	10.15	7.17	13.91
F. hanangensis	4310	Paratype	Mbulu	2	F?	80	3	skull & tissue					35.98	1.44	33.76	28.07	19.26	12.12	14.94	9.59	24.93	1.04	6.06	5.77	1.75	1.38	3.17	12.92	5.78	2.26	6.34	13.82	2.31	11.21	8.12	16.46
F. hanangensis	4311	Paratype	Mbulu	2	M	68	2	skull & tissue					36.85	1.51	31.18	27.73	19.22	11.02	15.40	9.65	24.39	1.15	6.31	5.70	1.89	1.73	3.60	12.31	6.13	2.60	8.63	14.53	2.05	10.89	7.54	15.33
F. hanangensis	4312	Paratype	Mbulu	2	M?	55	2	skull & tissue					33.43	1.46	28.96	25.42	17.88	10.79	13.74	9.34	22.87	0.96	6.01	5.23	1.77	1.34	3.40	12.13	6.06	2.35	8.19	14.99	1.91	10.29	7.10	14.03
F. hanangensis	4313	Paratype	Mbulu	2	F	47	1	skull & tissue					32.18	1.49	27.77	23.79	16.22	8.88	12.46	6.53	21.60	0.96	na	5.16	1.85	1.17	3.11	10.25	na	2.63	7.65	13.87	1.83	9.49	6.55	13.05
F. hanangensis	4314	Paratype	Mbulu	3	M	140		whole animal	134.5	12.1	22.8	24.0																								
F. hanangensis	4315	Paratype	Mbulu	3	M?	85	2	skull & tissue					36.19	1.45	31.29	26.79	19.19	11.20	15.07	7.92	24.95	0.92	6.09	5.36	1.95	1.60	3.12	12.31	6.45	2.21	8.26	14.42	2.05	8.72	6.92	15.83
F. hanangensis	4316	Paratype	Mbulu	3	F	54	1	skull & tissue					32.84	1.52	27.40	23.84	16.61	8.85	12.81	6.69	21.58	0.90	5.68	5.19	2.01	1.28	3.55	11.00	6.00	2.45	7.47	13.76	1.57	9.95	6.69	13.24
F. hanangensis	4317	Paratype	Mbulu	3	M?	65	3	skull & tissue					35.25	1.52	31.06	26.58	18.75	10.43	14.58	9.78	23.12	0.90	5.97	5.32	1.88	1.73	3.69	12.25	6.37	2.93	8.38	14.51	1.89	10.85	6.83	14.31
F. hanangensis	4318	Paratype	Mbulu	4	F	60		whole animal	109.8	12.2	21.6	21.6																								
F. hanangensis	4319	Paratype	Mbulu	4	M	100		whole animal	131.8	11.0	23.1	24.5																								
F. hanangensis	4320	Paratype	Mbulu	4	M	103	3	skull & tissue					38.15	1.45	33.50	39.15	20.67	12.79	16.01	10.96	26.26	1.26	6.32	5.61	1.92	1.58	3.88	12.89	6.06	2.30	8.82	15.10	2.07	11.30	7.56	16.89
F. hanangensis	4321	Paratype	Mbulu	4	M	85		whole animal	123.4	11.8	22.9	23.5																								
F. hanangensis	4322	Paratype	Mbulu	4	F	79	3	skull & tissue					36.27	1.47	32.21	28.19	19.78	11.18	14.83	10.32	24.70	0.87	6.04	5.28	1.85	1.54	3.84	12.97	6.04	3.01	8.51	14.79	1.96	11.17	7.09	15.70
F. hanangensis	4323	Paratype	Mbulu	4	F	75	3	skull & tissue					34.43	1.46	31.20	26.26	18.30	10.69	13.92	8.67	23.65	0.94	5.84	5.41	1.87	1.01	3.22	11.23	5.70	2.65	8.38	14.56	1.97	8.95	6.94	14.91
F. hanangensis	4324	Paratype	Mbulu	4	M	60	2	skull & tissue					35.49	1.48	31.69	26.68	18.31	9.95	14.31	10.02	23.94	0.97	5.87	5.29	1.80	1.33	3.55	11.53	5.94	2.85	7.49	14.18	1.74	11.00	6.50	14.64
F. hanangensis	4325	Paratype	Mbulu	4	M	35	1	skull & tissue					30.45	1.51	26.40	21.89	14.94	8.13	11.01	8.70	20.17	0.92	5.85	5.17	1.75	1.22	3.48	9.79	5.37	3.00	7.04	13.80	1.42	8.87	6.15	11.80
F. hanangensis	4326	Paratype	Mbulu	5	M	130	3	skull & tissue					39.37	1.37	36.52	31.02	21.28	12.75	16.67	8.01	28.83	0.80	6.48	5.77	2.06	2.22	4.46	14.64	5.88	2.75	8.93	14.95	2.46	10.26	8.96	18.78
F. hanangensis	4327	Paratype	Mbulu	5	M	75	1	skull & tissue					34.87	1.47	30.39	26.52	18.53	10.59	14.05	8.02	23.71	0.95	5.82	5.30	1.80	1.32	3.49	11.98	5.85	2.86	8.24	14.23	1.92	8.39	7.38	14.40
F. hanangensis	4328	Paratype	Mbulu	5	M	130		whole animal	133.6	9.4	23.2	–																								
F. hanangensis	4329	Paratype	Mbulu	5	M?	60	2	skull & tissue					33.22	1.48	29.06	25.03	17.67	9.53	12.91	7.88	22.49	0.83	6.15	5.48	1.92	1.31	4.83	12.15	6.44	2.79	8.05	13.66	1.70	8.44	7.48	13.78
F. hanangensis	4330	Paratype	Mbulu	5	na	53	1	skull & tissue					31.90	1.45	28.30	25.04	16.99	10.12	12.23	9.26	22.05	1.07	5.99	5.50	1.95	1.14	3.49	11.65	5.68	2.29	7.69	13.73	1.68	10.78	7.36	13.15
F. hanangensis	4331	Paratype	Mbulu	5	M	65	3	skull & tissue					35.41	1.47	31.52	27.34	18.85	10.03	13.96	8.22	24.15	0.76	6.47	5.55	1.87	1.84	3.51	12.64	6.31	2.92	7.82	15.19	1.91	9.32	7.08	15.77
F. hanangensis	4332	Paratype	Mbulu	5	M	35	2	skull & tissue					30.90	1.51	26.51	22.55	16.33	9.58	11.44	8.59	20.50	0.88	4.67	5.23	1.79	1.29	3.19	10.18	4.88	2.14	7.56	13.73	1.50	10.49	6.70	11.72
F. hanangensis	4333	Paratype	Mbulu	6	F	100	2	skull & tissue					37.51	1.42	34.19	29.39	20.76	12.28	16.20	10.26	26.40	0.93	5.94	5.39	1.98	1.73	3.95	13.73	5.82	2.91	8.48	14.49	2.26	9.73	7.73	17.51
F. hanangensis	4334	Paratype	Mbulu	6	M	140	4	skull & tissue					40.32	1.43	35.41	31.19	22.12	12.48	17.35	11.52	28.22	0.90	6.33	5.64	1.99	1.87	3.95	14.45	6.56	3.24	9.41	15.24	2.39	13.00	8.46	18.31
F. hanangensis	4335	QM sample	Mbulu	6	F	75		whole animal	122.9	12.7	21.7	22.0																								
F. hanangensis	4336	QM sample	Mbulu	7	M	55	1	skull & tissue					33.29	1.52	29.47	24.59	17.03	9.80	12.81	8.04	21.96	0.98	6.13	5.31	1.79	1.33	3.20	11.26	6.00	2.71	7.89	14.51	2.16	8.03	6.23	13.92
F. hanangensis	4337	QM sample	Mbulu	7	F	87		whole animal	126.7	10.0	21.6	21.6																								
F. hanangensis	4338	QM sample	Mbulu	7	M	130	3	skull & tissue					39.61	1.37	34.74	30.85	21.83	12.84	17.42	8.29	28.86	0.89	6.39	5.65	2.07	1.45	4.10	14.07	6.29	3.03	8.66	15.10	2.56	10.62	8.52	18.60
F. hanangensis	4339	QM sample	Mbulu	7	M	115	3	skull & tissue					37.85	1.42	32.08	29.16	20.48	12.34	15.91	8.15	26.71	1.09	6.34	5.67	2.18	1.35	3.47	13.38	5.83	3.02	8.94	15.27	2.86	9.65	8.33	17.68
								**Mean**	**115.64**	**11.08**	**22.35**	**22.61**	**35.19**	**1.47**	**31.02**	**27.17**	**18.65**	**10.70**	**14.25**	**8.87**	**24.01**	**0.97**	**6.04**	**5.43**	**1.90**	**1.46**	**3.57**	**12.22**	**6.03**	**2.66**	**8.09**	**14.39**	**1.98**	**10.05**	**7.26**	**15.14**
								**± SEM**	**6.45**	**0.40**	**0.23**	**0.39**	**0.51**	**0.01**	**0.52**	**0.67**	**0.35**	**0.26**	**0.33**	**0.23**	**0.47**	**0.03**	**0.07**	**0.04**	**0.02**	**0.05**	**0.09**	**0.25**	**0.08**	**0.06**	**0.13**	**0.11**	**0.07**	**0.22**	**0.14**	**0.38**
F. livinstoni	5208	Holotype	Ujiji	1	M	50	2	whole animal	115.4	8.9	22.0	–	33.14	1.49	30.11	25.30	17.66	9.53	13.02	7.79	22.27	0.92	5.60	4.99	1.80	1.54	3.36	10.66	5.59	2.83	8.13	14.10	2.07	9.72	6.99	14.04
F. livinstoni	5209	Paratype	Ujiji	1	F	35	1	skull & tissue					30.33	1.62	25.55	21.65	15.67	8.35	10.93	7.84	18.75	0.79	6.20	4.89	1.77	1.18	3.33	8.92	5.44	2.64	7.94	13.98	1.92	8.60	6.19	11.44
F. livinstoni	5210	Paratype	Ujiji	2?	F	80	4	skull & tissue					–	–	–	–	–	–	–	–	–	–	–	–	–	–	–	–	–	–	–	–	–	–	–	–
F. livinstoni	5211	Paratype	Ujiji	3?	M	42	1	skull & tissue					31.44	1.51	27.11	23.31	16.72	8.00	11.52	7.74	20.78	1.18	na	5.44	2.00	1.31	3.02	9.66	na	na	7.51	14.30	2.03	7.96	6.79	12.70
F. livinstoni	5212	Paratype	Ujiji	1	M	38	2	whole animal	120.0	7.3	20.4	20.4	31.32	1.51	27.49	22.91	15.99	8.40	12.25	7.83	20.70	1.00	5.10	5.21	1.87	1.45	3.32	9.74	5.61	2.59	7.38	14.16	2.05	8.07	6.38	12.44
F. livinstoni	5213	Paratype	Ujiji	4	M	52	2	whole animal	139.2	10.0	22.8	22.8	35.12	1.53	31.29	26.54	18.51	9.65	13.90	7.68	22.88	0.98	5.78	5.39	1.90	1.62	3.34	11.79	5.93	2.28	7.70	14.75	2.15	8.52	7.12	14.93
								**Mean**	**124.87**	**8.73**	**21.71**	**21.59**	**32.27**	**1.53**	**28.31**	**23.94**	**16.91**	**8.79**	**12.32**	**7.78**	**21.08**	**0.97**	**5.67**	**5.18**	**1.87**	**1.42**	**3.27**	**10.15**	**5.64**	**2.59**	**7.73**	**14.26**	**2.04**	**8.57**	**6.69**	**13.11**
								**± SEM**	**7.26**	**0.78**	**0.69**	**1.19**	**0.84**	**0.02**	**1.05**	**0.87**	**0.53**	**0.34**	**0.53**	**0.03**	**0.72**	**0.06**	**0.23**	**0.11**	**0.04**	**0.08**	**0.06**	**0.49**	**0.10**	**0.11**	**0.14**	**0.13**	**0.04**	**0.31**	**0.18**	**0.62**
F. whytei	3913	QM sample	Kigogo	1	F	101	1	skull & tissue	131.0	–	–	21.0	32.65	1.46	30.23	26.26	18.72	10.54	13.97	8.05	22.32	0.62	na	5.21	2.17	1.62	3.14	11.55	na	2.22	7.90	14.00	1.94	9.11	7.23	13.63
F. whytei	3915	QM sample	Kigogo	2	M	134	3	skull & tissue	153.0	–	–	25.0	40.24	1.44	35.72	32.95	22.69	13.26	18.14	9.17	27.99	0.93	6.32	5.87	2.32	1.85	4.04	15.05	6.95	2.43	8.86	14.90	2.45	10.50	9.17	18.23
F. whytei	3916	QM sample	Kigogo	3	F	124	3	skull & tissue	140.0	–	–	24.5	41.68	1.47	36.67	32.84	23.99	13.78	17.94	8.66	28.27	1.09	6.19	6.23	2.41	1.81	4.07	14.45	6.01	2.37	8.59	15.11	2.93	11.09	9.01	18.59
								**Mean**	**141.33**			**23.50**	**38.19**	**1.46**	**34.21**	**30.68**	**21.80**	**12.53**	**16.68**	**8.63**	**26.19**	**0.88**	**6.26**	**5.77**	**2.30**	**1.76**	**3.75**	**13.68**	**6.48**	**2.34**	**8.45**	**14.67**	**2.44**	**10.23**	**8.47**	**16.82**
								**± SEM**	**6.39**			**1.26**	**2.80**	**0.01**	**2.01**	**2.21**	**1.59**	**1.00**	**1.36**	**0.32**	**1.94**	**0.14**	**0.06**	**0.30**	**0.07**	**0.07**	**0.31**	**1.08**	**0.47**	**0.06**	**0.29**	**0.34**	**0.29**	**0.59**	**0.62**	**1.60**
