# Supplementary material for: Relic populations of Fukomys mole-rats in Tanzania: description of two new species F. livingstoni sp. nov. and F. hanangensis sp. nov
Source: PeerJ. 2017 Apr 27;5:e3214. doi: 10.7717/peerj.3214 (PMC5410139; doi:10.7717/peerj.3214)
Supplement: Table S2 — Results are displayed for both three species (F. livingstoni, F. hanangensis and F. whytei) and two species (F. livingstoni vs F. hanangensis) MANOVA. Significant differences are indicated in bold, and values remining significant after the post-hoc Tukey test and Bonferroni correction indicated as “yes” in the column “Signif.”. Diff refers to the difference in absolute values of the respective measurement (mm) between the first and second species listed (see Table S1). [file peerj-05-3214-s006.pdf]

Table S2. Summary statistics for craniometrics. Results are displayed for both three species (*F. livingstoni*, *F. hanangensis* and *F. whytei*) and two species (*F. livingstoni* vs *F. hanangensis*) MANOVA. Significant differences are indicated in bold, and values remining significant after the post-hoc Tukey test and Bonferroni correction indicated as "yes" in the column "Signif.". Diff refers to the difference in absolute values of the respective measurement (mm) between the first and second species listed (see Table S1).

		Three species MANOVA		Three species ANOVA with Tukey HSD									Two species MANOVA	
				F. livingstoni vs *F. hanangensis*			*F. whytei* vs *F. hanangensis*			*F. whytei* vs *F. livingstoni*			*F. liv.* vs *F. han.*	
	Craniometric measurement	$F_{2,28}$	P	Diff	P	Signif.	Diff	P	Signif.	Diff	P	Signif.	$F_{1,27}$	P
M1	greatest length of skull	7.67	**< 0.01**	-2.92	0.089	ns	3.00	0.187	ns	5.92	0.015	ns	4.3	**0.040**
M2	condylobasal length	5.76	**0.01**	-2.71	0.113	ns	3.19	0.142	ns	5.90	0.014	ns	3.23	0.080
M3	henselion-basion length	4.88	**0.02**	-3.23	0.129	ns	3.51	0.206	ns	6.74	0.024	ns	3.22	0.080
M4	henselion-palation length	9.38	**< 0.01**	-1.74	0.140	ns	3.15	0.021	ns	4.89	0.002	ns	3.66	0.070
M6	length of diastema	9.01	**< 0.01**	-1.91	0.015	ns	1.83	0.072	ns	3.74	0.001	ns	6.89	**0.010**
M7	distance between M1 and the foremost edge of the upper incisor	7.7	**< 0.01**	-1.92	0.067	ns	2.43	0.063	ns	4.36	0.004	ns	4.07	0.050
M8	smallest interorbital breadth	2.26	0.12	-	-	-	-	-	-	-	-	-	4.36	**0.040**
M9	zygomatic breadth on the zygomatic process of the squamosal	6.43	**0.01**	-2.93	0.043	ns	2.18	0.300	ns	5.12	0.016	ns	5.59	**0.030**
M10	smallest palatal breadth between first upper molars	0.32	0.73	-	-	-	-	-	-	-	-	-	0.36	0.550
M11	length of upper cheekteeth	2.14	0.14	-	-	-	-	-	-	-	-	-	3.22	0.080
M12	breadth of upper dental arch	13.48	**< 0.01**	-0.24	0.116	ns	0.34	0.068	ns	0.59	0.007	ns	8.31	**0.010**
M13	greatest breadth of first upper molar	17.17	**< 0.01**	-0.03	0.811	ns	0.40	<0.001	**yes**	0.43	0.000	**yes**	1.29	0.270
M14	smallest breadth of zygomatic plate	1.83	0.18	-	-	-	-	-	-	-	-	-	0.03	0.860
M15	greatest breadth of nasals	2.11	0.14	-	-	-	-	-	-	-	-	-	1.31	0.260
M16	greatest length of nasals	9.66	**< 0.01**	-2.07	0.007	ns	1.46	0.169	ns	3.53	0.002	ns	9.41	**< 0.01**
M17	length of lower cheekteeth	3.32	0.05	-	-	-	-	-	-	-	-	-	3.72	0.060
M18	greatest breadth of the choanae	0.69	0.51	-	-	-	-	-	-	-	-	-	0.18	0.670
M19	length of auditory bulla	1.48	0.25	-	-	-	-	-	-	-	-	-	0.87	0.360
M20	greatest breadth of braincase	1.38	0.27	-	-	-	-	-	-	-	-	-	0.31	0.580
M21	depth of upper incisors	4.3	**0.02**	0.06	0.926	ns	0.46	0.075	ns	0.40	0.241	ns	0.11	0.740
M22	mediosagittal projection of rostrum height at anterior border of first upper molars	3.34	0.05	-	-	-	-	-	-	-	-	-	5.18	**0.030**
M23	greatest rostrum breadth	8.16	**< 0.01**	-0.56	0.272	ns	1.21	0.027	ns	1.78	0.006	ns	2.56	0.120
M24	distance between the extreme points of coronoid and angular processes of mandibular	5.14	**0.01**	-2.03	0.101	ns	1.67	0.354	ns	3.71	0.038	ns	3.82	0.060
